# Superior Mesenteric Vein Thrombosis in a Young Lowlander at High Altitude: A Case of Delayed Diagnosis Due to Isolated Fever

**DOI:** 10.7759/cureus.105207

**Published:** 2026-03-14

**Authors:** Shruti Chauhan, Sanjeev Kumar, Sankalp Singh, Narendra Jain

**Affiliations:** 1 General Medicine, Military Hospital, Kargil, IND; 2 Surgery, INHS Asvini, Mumbai, IND; 3 Radiation Oncology, Army Hospital Research and Referral, Delhi, IND; 4 Radiation Oncology, Military Hospital, Kargil, IND; 5 Radiodiagnosis, Command Hospital, Panchkula, IND

**Keywords:** bowel ischemia, high altitude, lowlander, mesenteric vein thrombosis, young adult

## Abstract

Superior mesenteric vein thrombosis (SMVT) is an uncommon but potentially life-threatening condition, particularly in high-altitude environments where hypoxia induces a prothrombotic state by elevating hematocrit, promoting endothelial dysfunction, and upregulating procoagulant factors, thereby substantially increasing the risk of SMVT and other thrombotic diseases. We report a rare clinical presentation of SMVT in a 30-year-old male military lowlander deployed at 17,000 ft for three months, who initially presented with isolated low-grade fever, leading to a delayed diagnosis (11 days to abdominal pain onset and 20 days to imaging). Contrast-enhanced computed tomography (CECT) confirmed SMVT with mesenteric fat stranding, but no bowel ischemia. This case underscores the importance of maintaining a high index of suspicion for thromboembolic disorders in high-altitude personnel, even with nonspecific symptoms, such as fever.

## Introduction

Superior mesenteric vein thrombosis (SMVT) is an uncommon condition with a high risk of mortality if untreated. The incidence in the general population is low, estimated at about 2.7 cases per 100,000 people annually. It accounts for 6%-9% of all cases of mesenteric ischemia and primarily affects middle-aged or older adults with hypercoagulable states, malignancy, or intra-abdominal pathology [1,2]. SMVT obstructs venous return, leading to intestinal congestion and edema due to impaired blood drainage rather than arterial occlusion alone. High-altitude exposure, due to hypoxia-induced polycythemia, endothelial dysfunction, and dehydration, increases the incidence of venous thromboembolism by 2- to 30-fold in lowlanders [3,4]. Although cases of SMVT occurring at high altitude have been reported, the available literature remains limited.

Clinically, SMVT may present with acute or insidious symptoms, including abdominal pain, diarrhea, melena, nausea, vomiting, and hematemesis, often with subtle clinical signs such as abdominal guarding. Fever, when present, usually suggests an underlying infection, such as pylephlebitis, acute diverticulitis, or appendicitis, and is almost always associated with abdominal pain or discomfort. Fever may also result from mesenteric inflammation caused by tissue hypoxia, which can contribute to diagnostic delay. Routine ultrasound in early cases is often unremarkable, and low clinical suspicion in such situations may further delay diagnosis [1]. Elevation of D-dimer levels is a sensitive but non-specific biomarker for thromboembolic occlusion; however, persistently normal values on serial testing may help exclude SMVT [1]. Contrast-enhanced computed tomography (CECT) in the portal venous phase is the imaging modality of choice, demonstrating filling defects, bowel wall edema, collateral vessels, bowel dilatation, pneumatosis, or ascites [5].

Treatment depends on disease severity and ranges from anticoagulation to surgery. Patients without clinical signs of peritonitis on serial abdominal examinations and without radiological evidence of intestinal ischemia or infarction can be managed conservatively with anticoagulation, usually starting with low-molecular-weight heparin followed by an oral vitamin K antagonist (VKA) or a direct oral anticoagulant (DOAC) [6].

We describe an uncommon presentation of SMVT in a young lowlander staying at an extreme-altitude region who initially presented with isolated low-grade fever, which delayed further evaluation until abdominal pain developed. This scenario led to a significant diagnostic delay and, to our knowledge, has not been previously reported in the SMVT literature.

Written informed consent was obtained from the patient for publication of this case report and the accompanying images.

## Case presentation

A 30-year-old male military personnel, a lowlander with no known comorbidities except active smoking (5 pack-years), was deployed at an altitude of 17,000 ft for three months. He presented in January 2026 with a low-grade continuous fever (up to 100.8 °F), which was fully relieved by antipyretics. No other symptoms, such as cough, dyspnea, headache, or gastrointestinal complaints, were reported. General physical and systemic examinations were normal at admission, and no serial abdominal examinations were performed during the febrile phase. He was managed symptomatically for a presumed viral illness, with no further evaluation at that time.

**Figure 1:**
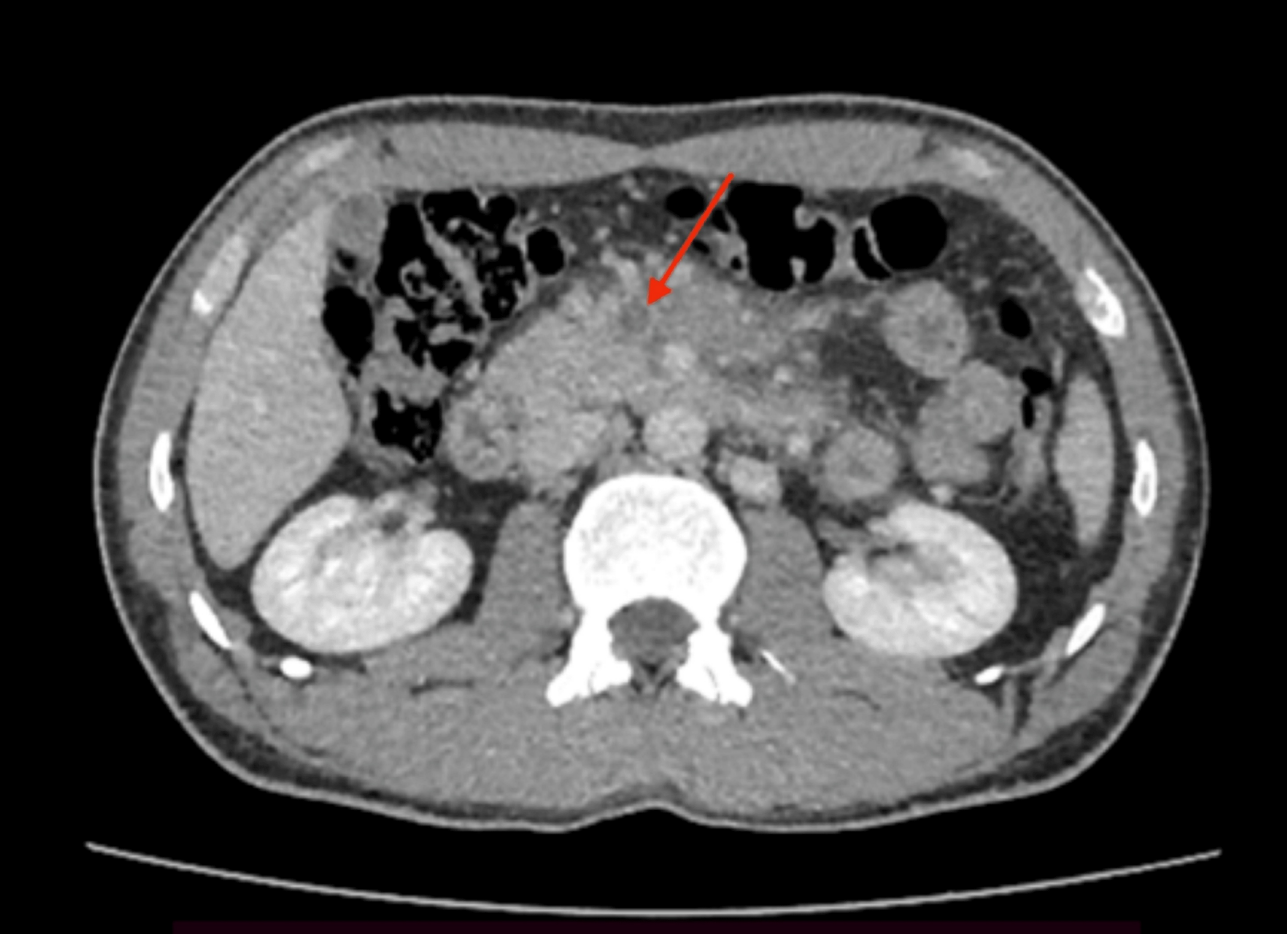
Axial abdominal CECT in the portal venous phase showing a filling defect in the superior mesenteric vein (arrow) CECT: contrast-enhanced computed tomography.

**Figure 2:**
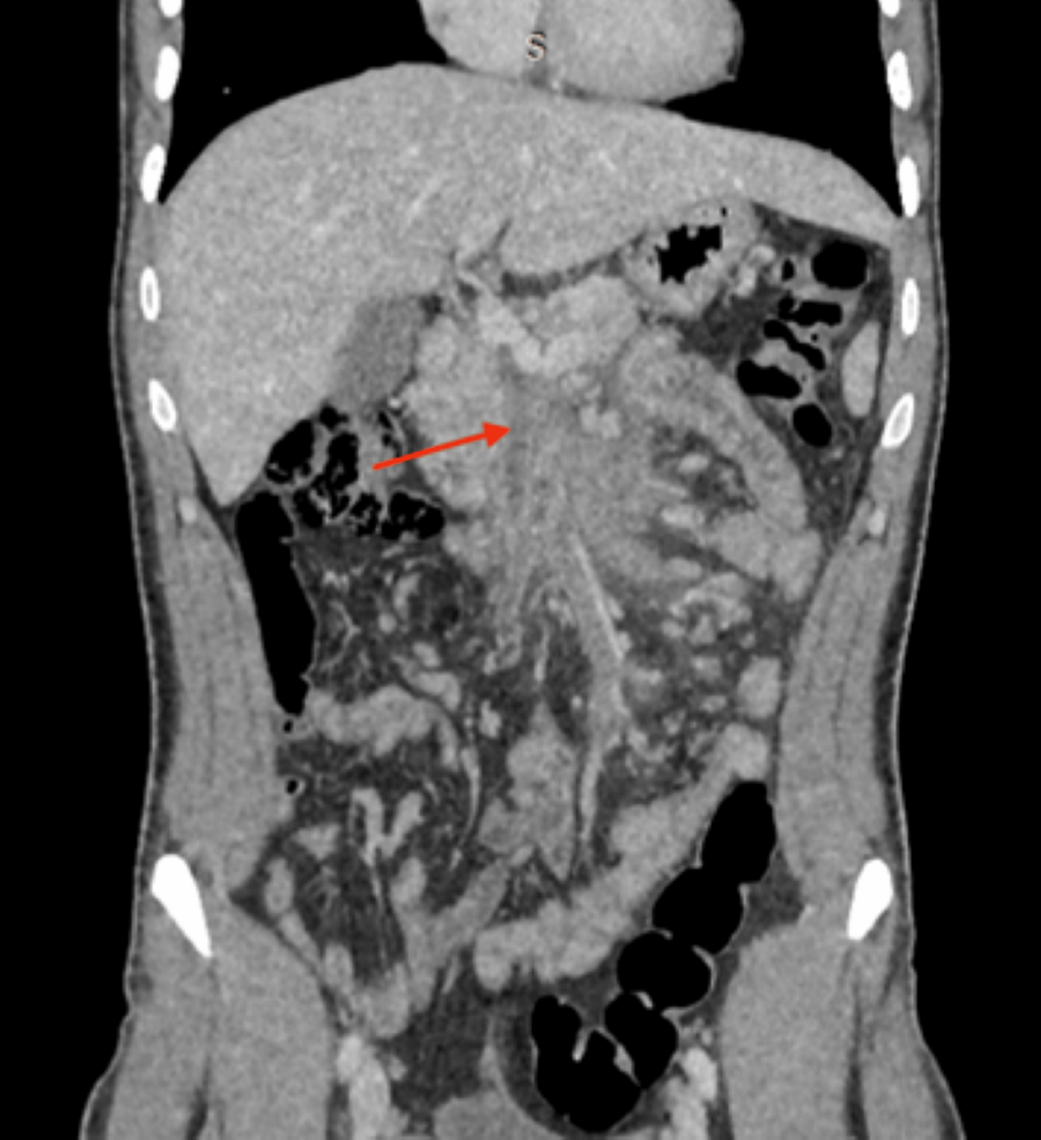
Coronal reformat demonstrating the extent of the thrombus, with associated mesenteric fat stranding (arrow)

Eleven days after the initial presentation, he developed non-radiating periumbilical abdominal pain without nausea, vomiting, constipation, or melena. Examination revealed guarding and tenderness in the periumbilical region, with no other significant systemic findings (stable vital signs, no organomegaly, and no signs of peritonitis). Baseline hematological and biochemical parameters were within normal limits (Table 1). D-dimer levels were elevated at 1640 ng/mL (normal value <500 ng/mL). Abdominal ultrasonography (performed without color-flow Doppler) was unremarkable. Contrast-enhanced computed tomography (CECT) of the abdomen, performed one week later (20 days from the first presentation), revealed SMVT with associated mesenteric fat stranding and minimal free fluid in the pelvis, but no evidence of bowel ischemia or infarction (Figures 1, 2).

**Table 1:** Laboratory investigations SGOT: serum glutamic-oxaloacetic transaminase, SGPT: serum glutamic-pyruvic transaminase.

Parameter	Value	Reference range (typical)
Hematological		
Hemoglobin (Hb)	16.8 g/dL	13.5-17.5 g/dL (males)
Total leukocyte count (TLC)	8.2 × 10³/μL	4-11 × 10³/μL
Platelets	280 × 10³/μL	150-450 × 10³/μL
Neutrophils	71%	40%-75%
Lymphocytes	20%	20%-45%
Monocytes	5%	2%-10%
Eosinophils	4%	1%-6%
Blood sugar		
Random blood sugar (RBS)	101 mg/dL	<140 mg/dL (non-fasting)
Renal function tests (RFT)		
Urea	25 mg/dL	15-40 mg/dL
Creatinine	0.9 mg/dL	0.6-1.2 mg/dL
Electrolytes		
Sodium (Na)	137 mEq/L	135-145 mEq/L
Potassium (K)	4.3 mEq/L	3.5-5.0 mEq/L
Chloride (Cl)	101 mEq/L	98-106 mEq/L
Liver function tests (LFT)		
Total bilirubin	1.5 mg/dL	0.3-1.2 mg/dL
Direct bilirubin	0.7 mg/dL	0-0.3 mg/dL
Aspartate aminotransferase (AST; SGOT)	16 IU/L	10-40 IU/L
Alanine transaminase (ALT; SGPT)	22 IU/L	10-40 IU/L
Alkaline phosphatase (ALP)	154 IU/L	44-147 IU/L
Amylase	46 IU/L	25-125 IU/L
Lipase	37 IU/L	13-60 IU/L
Other biochemical		
D-dimer	1640 ng/mL	<500 ng/mL
Urine		
Routine microscopy (RE/ME)	No abnormality detected (NAD)	-
Coagulation		
Prothrombin time (PT)	16 sec	11-16 sec
International normalized ratio (INR)	1.1	0.8-1.2

He was started on low-molecular-weight heparin (Enoxaparin 60 mg subcutaneously twice daily) with close monitoring of his condition. The patient’s symptoms improved within 48 hours. Serial abdominal examinations showed resolution of tenderness and improvement in guarding. Anticoagulation therapy was continued. The patient was air-evacuated to a tertiary care center at a lower altitude. At the end of the first month of anticoagulation, repeat CECT of the abdomen showed partial recanalization of the SMVT, with resolution of mesenteric fat stranding and no evidence of bowel ischemia. The patient remained asymptomatic, with normalized D-dimer levels (320 ng/mL), and was transitioned to oral rivaroxaban 20 mg daily. Table 2 displays the timeline of the relevant events in this case.

**Table 2:** Patient's timeline of events CECT: contrast-enhanced computed tomography, SMVT: superior mesenteric vein thrombosis.

Timeline	Event/symptom	Investigations/management
Oct 2025-Jan 2026	Deployed at 17,000 ft for three months; asymptomatic except smoking history	None
Jan 2026	Low-grade fever (up to 100.8 °F), relieved by antipyretics	Symptomatic (presumed viral); no further evaluation
After 11 days of initial presentation	Periumbilical abdominal pain, guarding/tenderness	Labs normal except D-dimer 1640 ng/mL; US unremarkable
After 20 days of initial presentation	Persistent symptoms	CECT: SMVT with fat stranding, no ischemia; started enoxaparin 60 mg SC BD
After 22 days of initial presentation	Symptom improvement in 48 h, resolution of tenderness, and improvement in guarding	Air-evacuated to tertiary center
One month post-anticoagulation	Patient remained asymptomatic	Repeat CECT: partial recanalization of the SMVT, resolution of mesenteric fat stranding, no ischemia. Normalized D-dimer (320 ng/mL); transitioned to oral rivaroxaban 20 mg daily

## Discussion

High-altitude exposure induces endothelial dysfunction, polycythemia, and hypercoagulability via hypoxia-inducible factors, increasing the risk of venous thromboembolism in lowlanders [7,8]. Although rare (incidence 2.7/100,000), SMVT may present insidiously, with fever reported in 20%-50% of cases due to mesenteric inflammation, thereby mimicking common infections [1,7]. In this patient, three months at 17,000 ft likely exacerbated smoking-induced endothelial damage (5 pack-years) and dehydration risk in a deployment setting, despite normal hemoglobin levels indicating no overt polycythemia. In this case, the 11-day delay from fever onset to abdominal pain (and 20 days to imaging) resulted from attributing the fever to a benign viral etiology, a common pitfall in resource-limited high-altitude settings.

This case highlights thromboembolic disorders as an important differential diagnosis for isolated fever in high-altitude military personnel, especially in smokers or lowlanders. Early D-dimer screening (>500 ng/mL, indicating the need for imaging) and a low threshold for CECT can prevent progression to bowel infarction, which carries a mortality of up to 20%-50% if untreated [5]. Management decisions, particularly the need for surgical intervention, are guided primarily by the patient’s hemodynamic stability and the presence of peritoneal signs. Our case closely resembles the report by Anand et al. (2001), which described insidious abdominal pain due to high-altitude mesenteric venous thrombosis after prolonged exposure and delayed diagnosis, but differs in that fever was the initial presentation [9]. Normal hemoglobin levels in our patient suggest that endothelial dysfunction and hypercoagulability predominated, possibly worsened by smoking and dehydration [9]. Prophylactic measures, such as adequate hydration, smoking cessation, and graded acclimatization, are therefore essential. Other markers of thrombosis, including thrombin-antithrombin complex (TAT), thrombomodulin (TM), and tissue plasminogen activator inhibitor complex (t-PAIC), may also be elevated, although they remain non-specific and offer no clear clinical advantage over D-dimer [10]. A complete thrombophilia workup should be considered in all patients to rule out underlying coagulation disorders, especially in younger individuals.

A limitation of this report is that a full thrombophilia workup (protein C, protein S, antithrombin III, and factor V Leiden) was not performed due to resource limitations at the remote location, although the patient was advised to undergo testing before redeployment to a high-altitude region. In addition, as this is a single case report, the observations and conclusions may not be generalizable to all patients presenting with similar symptoms in high-altitude environments.

## Conclusions

SMVT at extreme altitudes can present in many unusual ways, which can delay diagnosis. Clinicians must prioritize thromboembolic evaluation in such scenarios to avoid such complications (e.g., checking D-dimer levels in cases of unexplained fever at high altitude). Additionally, incorporating thrombophilia screening into pre-deployment medical checkups for lowlanders like military personnel posted to high altitudes for extended periods is advisable. This proactive step helps identify at-risk individuals early, enabling tailored prophylaxis like anticoagulants or compression therapy before ascent. Ultimately, heightened vigilance and standardized protocols can safeguard personnel in these challenging environments.
